# Deficiency in non-classical major histocompatibility class II-like molecule, H2-O confers protection against *Staphylococcus aureus* in mice

**DOI:** 10.1371/journal.ppat.1012306

**Published:** 2024-06-06

**Authors:** Emily Cullum, Yunys Perez-Betancourt, Miaomiao Shi, Eirinaios Gkika, Olaf Schneewind, Dominique Missiakas, Tatyana Golovkina

**Affiliations:** 1 Department of Microbiology, University of Chicago, Chicago, Illinois, United States of America; 2 Committee on Immunology, University of Chicago, Chicago, Illinois, United States of America; 3 Howard T. Ricketts Laboratory, University of Chicago, Chicago, Illinois, United States of America; 4 Committee on Microbiology, University of Chicago, Chicago, Illinois, United States of America; 5 Committee on Genetics, Genomics and System Biology, University of Chicago, Chicago, Illinois, United States of America; University of Illinois at Chicago College of Medicine, UNITED STATES

## Abstract

*Staphylococcus aureus* is a human-adapted pathogen that replicates by asymptomatically colonizing its host. *S*. *aureus* is also the causative agent of purulent skin and soft tissue infections as well as bloodstream infections that result in the metastatic seeding of abscess lesions in all organ tissues. Prolonged colonization, infection, disease relapse, and recurrence point to the versatile capacity of *S*. *aureus* to bypass innate and adaptive immune defenses as well as the notion that some hosts fail to generate protective immune responses. Here, we find a genetic trait that provides protection against this pathogen. Mice lacking functional H2-O, the equivalent of human HLA-DO, inoculated with a mouse-adapted strain of *S*. *aureus*, efficiently decolonize the pathogen. Further, these decolonized animals resist subsequent bloodstream challenge with methicillin-resistant *S*. *aureus*. A genetic approach demonstrates that T-cell dependent B cell responses are required to control *S*. *aureus* colonization and infection in H2-O-deficient mice. Reduced bacterial burdens in these animals correlate with increased titers and enhanced phagocytic activity of *S*. *aureus*-specific antibodies. H2-O negatively regulates the loading of high affinity peptides on major histocompatibility class II (MHC-II) molecules. Thus, we hypothesize that immune responses against *S*. *aureus* are derepressed in mice lacking H2-O because more high affinity peptides are presented by MHC-II. We speculate that loss-of-function HLA-DO alleles may similarly control *S*. *aureus* replication in humans.

## Author summary

Humans develop antibodies upon exposure to *Staphylococcus aureus* but seroconversion does not necessarily protect against invasive diseases or their recurrence as documented by the complexity in treating such infections both in hospitals and communities. Colonization constitutes the highest risk factor for disease-causing infections. Yet, the number of infections is relatively low considering that all newborns are exposed to *S*. *aureus* at birth and the bacterium continues to colonize the nares of about a third of the adult population. Here we show that changes in the major histocompatibility class II (MHC-II) pathway that selects pathogen-specific antigens, result in enhanced protective immune responses against *S*. *aureus* in animals. We speculate that specific alleles of the genes involved in the MHC-II pathway may underlie a range of protective immune responses such that not all individuals exposed to *S*. *aureus* are at the same risk for infection.

## Introduction

Pathogens evolve successful strategies to evade defense mechanisms of their hosts. *Staphylococcus aureus* is a prime example of such a pathogen: it colonizes the human nares and skin, causes frequent skin, soft tissue, and bloodstream infections [1,2], and is adaptable to other hosts [3,4]. While colonization is innocuous and occurs at birth [5,6], it represents the highest risk for infection as disease isolates are typically the same as those cultured from the skin and nares of the host [7–10]. Despite the elicitation of *S*. *aureus*-specific antibodies, colonization persists and most studies point to the notion that these antibodies do not protect against invasive disease [8,10–14]. A number of reasons may account for this lack of protection: (i) *S*. *aureus* has evolved many mechanisms to escape the activity of antibodies [2]; (ii) the constant exposure to *S*. *aureus* may promote isotype switching and changes in glycosylation patterns of antibodies that preempt their engagement in adequate effector activities [14,15]; (iii) constant exposure may select for the presentation of non-protective epitopes of immunodominant antigens [16–18]; (iv) B and T cell superantigens (SAgs) secreted by *S*. *aureus* may skew the specificity of antibodies and T cells [19–21].

Several observations suggest that susceptibility to *S*. *aureus* is influenced by the genetic make-up of the host. For example, the major histocompatibility complex class II (MHC-II) locus has been proposed to act as a genetic determinant of susceptibility to *S*. *aureus* infection in humans [22,23]. This may in part be accounted for by the function of T cell-specific SAgs, which have preferential binding for specific MHC-II alleles as nasal carriers inherit specific HLA class II alleles [24]. However, there are over 100 genes mapped within the MHC locus in addition to MHC-II genes and all are polymorphic with half participating in immune responses. Because of the low frequency of recombination within the MHC region [25], the direct identification of genes that may modulate host-*S*. *aureus* interactions is challenging.

Previously we found that mice from the I/LnJ strain produce neutralizing antibodies against mouse mammary tumor virus (MMTV) [26,27]. These neutralizing antibodies, generated in a CD4 T cell-dependent fashion [28], coat viral particles rendering MMTV non-infectious [26,27]. This mechanism of resistance has been explained by the loss of H2-Ob (*Ob*) function and is not a peculiarity of I/LnJ animals, as MMTV-susceptible C57BL/6J (B6J) mice rendered *Ob*-deficient also produced virus-neutralizing antibodies [29]. *Ob* encodes the beta subunit (Oβ) of the non-classical MHC-II-like αβ heterodimer (H2-O in mice and HLA-DO or DO in humans) and is expressed in B cells, dendritic and thymic epithelial cells [29–33]. In humans, the non-classical αβ heterodimer is encoded by genes HLA-DOA (DOA) and HLA-DOB (DOB). Specific alleles of DOA and DOB with reduced- or loss-of-functions have been linked to resolution of infection with hepatitis B and C viruses [29,34] which require the early presence of broadly neutralizing antibodies [35–37]. The elicitation of neutralizing antibodies in the absence of H2-O has been explained by the uninhibited function of H2-M [29]. H2-M (HLA/DM or DM) catalyze peptide loading of MHC-II molecules in late endosomes and lysosomes of antigen presenting cells by replacing the MHC-II-associated invariant chain peptides with high-affinity, pathogen-derived peptides [38–45]. As H2-O inhibits H2-M function, its expression leads to a small but significant decrease of high affinity peptides presented by MHC-II molecules [43]. Conversely, the lack of H2-O/DO-mediated inhibition of H2-M/DM results in increased numbers of high affinity peptides presented by MHC-II molecules [43].

Since both the Oα and Oβ chains of H2-O/DO are encoded within the MHC-II locus, we wondered whether the loss of functional alleles influences the susceptibility or resistance toward *S*. *aureus*. Using a mouse model of colonization, we observed that animals with deficiency in H2-O cleared *S*. *aureus*. Successful decolonization required an intact T cell-dependent B cell response and directly correlated with increased titers of *S*. *aureus*-specific antibodies. Furthermore, H2-O-deficient mice also cleared the mouse common commensal, *Staphylococcus xylosus*.

## Results

### Rapid *S*. *aureus* decolonization is observed in H2-O-deficient animals

Recently, we developed a model of *S*. *aureus* nasopharyngeal colonization in mice to better understand bacterial and host factors that contribute to persistent host-bacterial interactions [3]. This model takes advantage of the mouse-adapted WU1 variant isolated from a mouse colony at Washington University [3]. When the variant is inoculated intranasally, wild type B6J mice remain colonized as monitored by plating nasopharyngeal swabs and fecal material on mannitol salt agar in weekly intervals [3]. This model is unlike other models that require pretreatment of animals with antibiotics for the transient colonization with human nosocomial or clinical isolates of *S*. *aureus*. B6J mice remain persistently colonized with WU1 provided that the bacterium produces Staphylococcal protein A (SpA), a factor that alters B cell responses [3,19,20].

**Fig 1:**
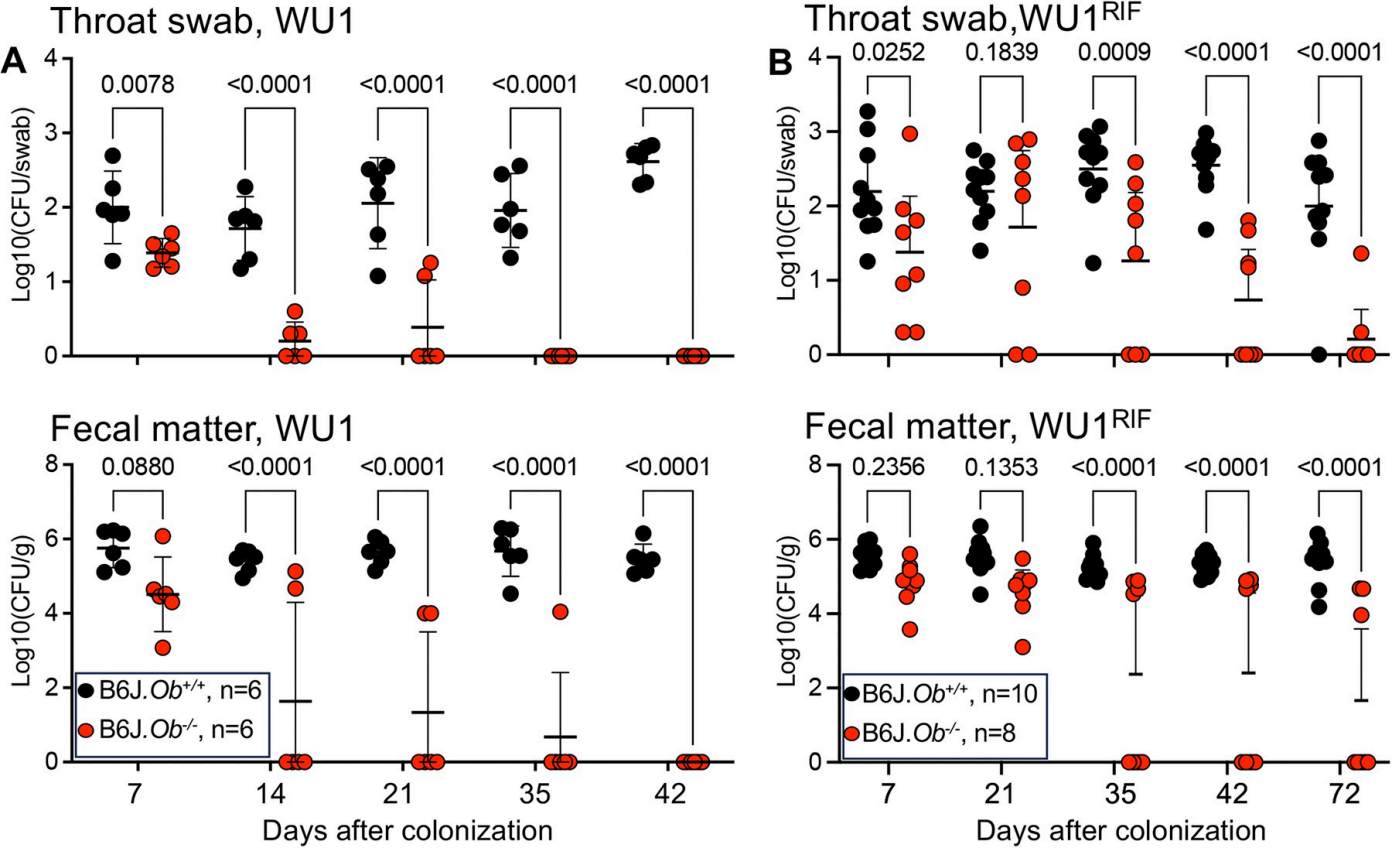
H2-O-deficient B6J mice decolonize *S*. *aureus*. Eight-week-old B6J.*Ob*^*+/+*^ and B6J.*Ob*^*-/-*^ mice were colonized intranasally with 10^8^ CFUs of WU1 **(A)** or 10^8^ CFUs of WU1^Rif^ **(B)** and monitored for colonization weekly by plating throat swabs **(A** and **B, top panels)** and fecal matter **(A** and **B, bottom panels)** on mannitol salt agar **(A)** or on rifampicin containing tryptic soy agar **(B)**. Mice with different genotypes were co-housed in the same cages. Data are presented as median ± 95% confidence interval. Significance was calculated using two-way ANOVA tests with multiple comparisons. n, number of mice used. Each dot represents a mouse. Males and females were used at 50:50 ratio. CFU, colony forming unit. Fecal and throat cultures performed on mice prior to inoculation of WU1 were negative for *S*. *aureus* species.

**Fig 2:**
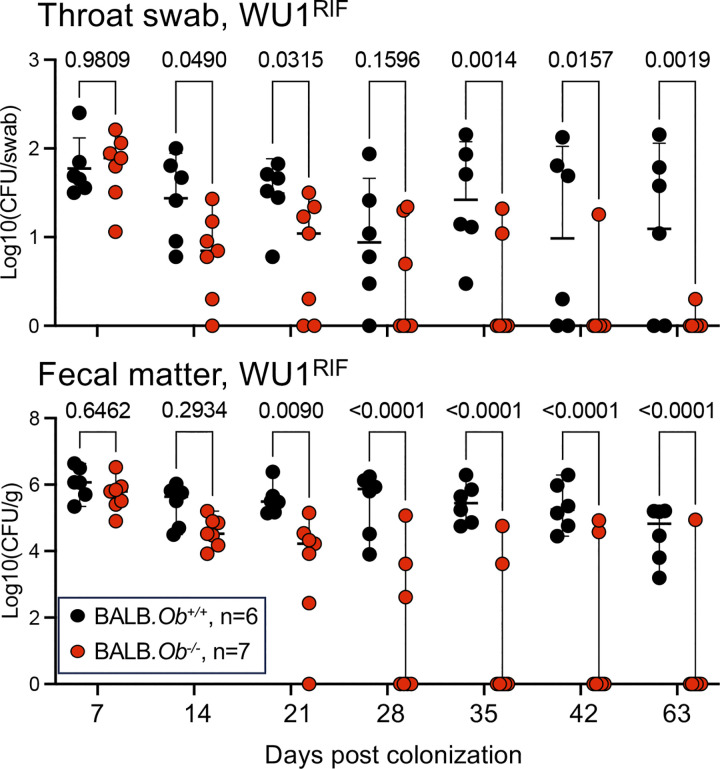
H2-O-deficient BALB/cJ mice decolonize *S*. *aureus*. Eight-week-old *Ob*^*+/+*^ and *Ob*^*-/-*^ BALB/cJ mice were colonized intranasally with 10^8^ CFUs of WU1^Rif^ and monitored for colonization weekly by plating throat swabs **(top panel)** and fecal matter **(bottom panel)**. Data are presented as median ± 95% confidence interval. Significance was calculated using two-way ANOVA tests with multiple comparisons. n, number of mice used. Each dot represents a mouse. Males and females were used at 50:50 ratio. CFU, colony forming units. Fecal and throat cultures performed on mice prior to inoculation of WU1 were negative for *S*. *aureus* species.

To test whether the MHC-II antigen presentation pathway and specifically H2-O play a role in controlling *S*. *aureus*, B6J.*Ob*^*+/+*^ and B6J.*Ob*^*-/-*^ mice were colonized with WU1 and bacterial loads in throat and feces were recorded weekly over the course of 7 weeks. Mice were scored as decolonized when both nasopharyngeal swabs and fecal matters were free of *S*. *aureus* following plating on mannitol salt agar. Both B6J.*Ob*^*+/+*^ and B6J.*Ob*^*-/-*^ animals carried similar loads of bacteria one week post inoculation; however, while B6J.*Ob*^*+/+*^ mice remained colonized, *Ob*^*-/-*^ animals cleared the bacteria within 5 weeks (Fig 1A). A rifampicin-resistant WU1 variant (WU1^Rif^) that carries a point mutation in *rpoB*, encoding the β subunit of RNA polymerase [46], was also used for colonization (Fig 1B). This was performed because plating on mannitol salt agar revealed the presence of both pigmented *S*. *aureus* and a non-pigmented Gram-positive organism which was subsequently identified as *S*. *xylosus* (see below). As with strain WU1, B6J.*Ob*^*-/-*^ cleared WU1^Rif^ in approximately 5 weeks while B6J.*Ob*^*+/+*^ animals remained colonized (Fig 1B). To examine whether loss of H2-O promotes decolonization of *S*. *aureus* independently of the mouse genetic background, BALB/cJ H2-O-deficient (BALB.*Ob*^*-/-*^) animals were also used. BALB.*Ob*^*+/+*^ mice were stably colonized with WU1^Rif^ throughout the duration of the experiment (Fig 2). Similar to B6J.*Ob*^*-/-*^ mice, more than 70% of BALB.*Ob*^*-/-*^ animals cleared *S*. *aureus* within 5 weeks post colonization (Fig 2). Therefore, we concluded that H2-O deficiency correlated with *S*. *aureus* decolonization in animals irrespectively of the genetic background. The notion that, when observed, clearance occurred approximately 5 weeks post colonization agrees with a role of H2-O within the MHC-II antigen presentation pathway specifically, the activation of adaptive immune responses.

### H2-O-deficient animals clear *S*. *xylosus*

**Fig 3:**
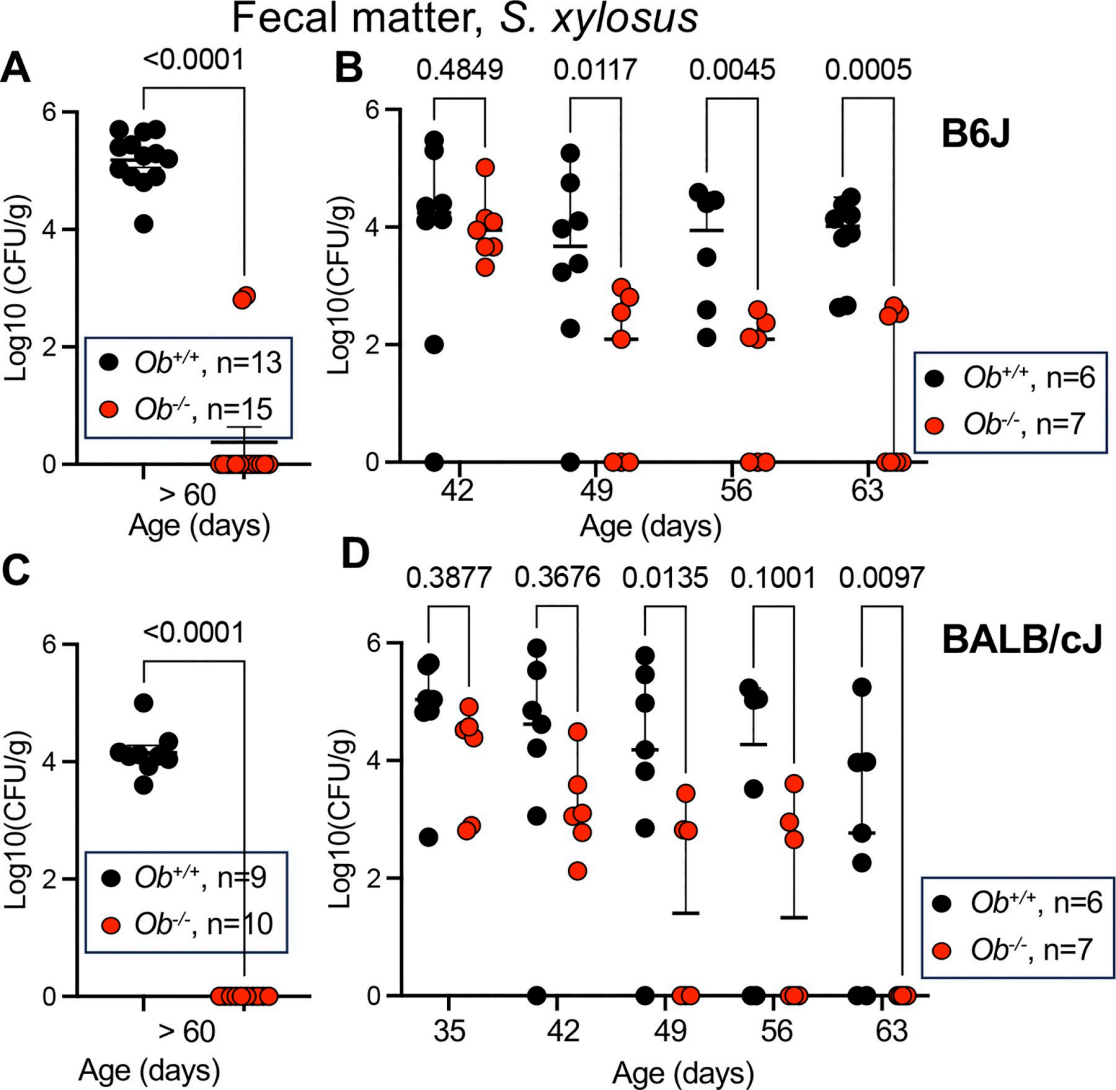
H2-O-deficient mice decolonize a commensal bacterium, *S*. *xylosus*. Colonies of approximately 60-day old *Ob*^*-/-*^ and *Ob*^*+/+*^ B6J **(A)** and BALB/cJ **(C)** mice born at the University of Chicago were screened by plating fecal matter on mannitol salt agar plates. All colonies looked identical on plate and the species was identified as *S*. *xylosus* using 16S RNA-specific primers. **(B)** B6J.*Ob*^*-/-*^and B6J.*Ob*^*+/+*^ were foster-nursed by *S*. *xylosus*-positive B6J.*Ob*^*+/+*^ mothers and **(C)** BALB/c.*Ob*^*-/-*^ and BALB/c.*Ob*^*+/+*^ were foster-nursed by *S*. *xylosus*-positive BALB.*Ob*^*+/+*^ mothers. All animals were monitored for colonization weekly by plating fecal matter on mannitol salt agar but data are shown starting at weeks 6 (**B**) and 5 (**D**) after birth. Significance was determined using unpaired *t* test (**A** and **C**) and two-way ANOVA tests with multiple comparisons (**B** and **D**). Data are presented as mean ± SEM (**A** and **C**) and as median ± 95% confidence interval (**B** and **D**). Males and females were used at 50:50 ratio. CFU, colony forming unit.

As noted above, plating on mannitol salt agar revealed the presence of non-pigmented colonies that acidified the medium as a result of metabolic activity. To rigorously establish the presence and identity of this bacterium, fecal samples of approximately 60-day old naïve B6J and BALB/c mice were plated on mannitol salt agar (Fig 3A and 3C). Colonies were observed in fecal samples of all wild type animals but only in two of fifteen B6J.*Ob*^*-/-*^ and none of ten BALB.*Ob*^*-/-*^ mice (Fig 3A and 3C). All colonies looked identical on plate. Forty-five colonies were selected from 4 different cages per mouse strain and PCR products were obtained using 16S RNA specific-primers [47]. DNA sequencing identified a single species, *S*. *xylosus*.

To ensure the H2-O-deficient mice could efficiently decolonize *S*. *xylosus* when they are exposed to the microbe, we foster-nursed newborn *Ob*^*-/-*^ mice by *Ob*^*+/+*^ mothers of the same background (Fig 3B and 3D). Fecal contents of fostered animals were monitored for the presence of *S*. *xylosus*. Bacterial counts decreased significantly in *Ob*^*-/-*^ mice of both backgrounds but not in *Ob*^*+/+*^ mice over time (Fig 3B and 3D). Decolonization became apparent at approximately 7 weeks of age. Thus, H2-O-deficient mice became colonized with both *S*. *aureus* and *S*. *xylosus* but successfully cleared these species in a manner coinciding with the development of T-cell-dependent Ab responses in mice [48,49].

### Clearance of *S*. *aureus* in *Ob*^*-/-*^ mice requires B and T cell functions

**Fig 4:**
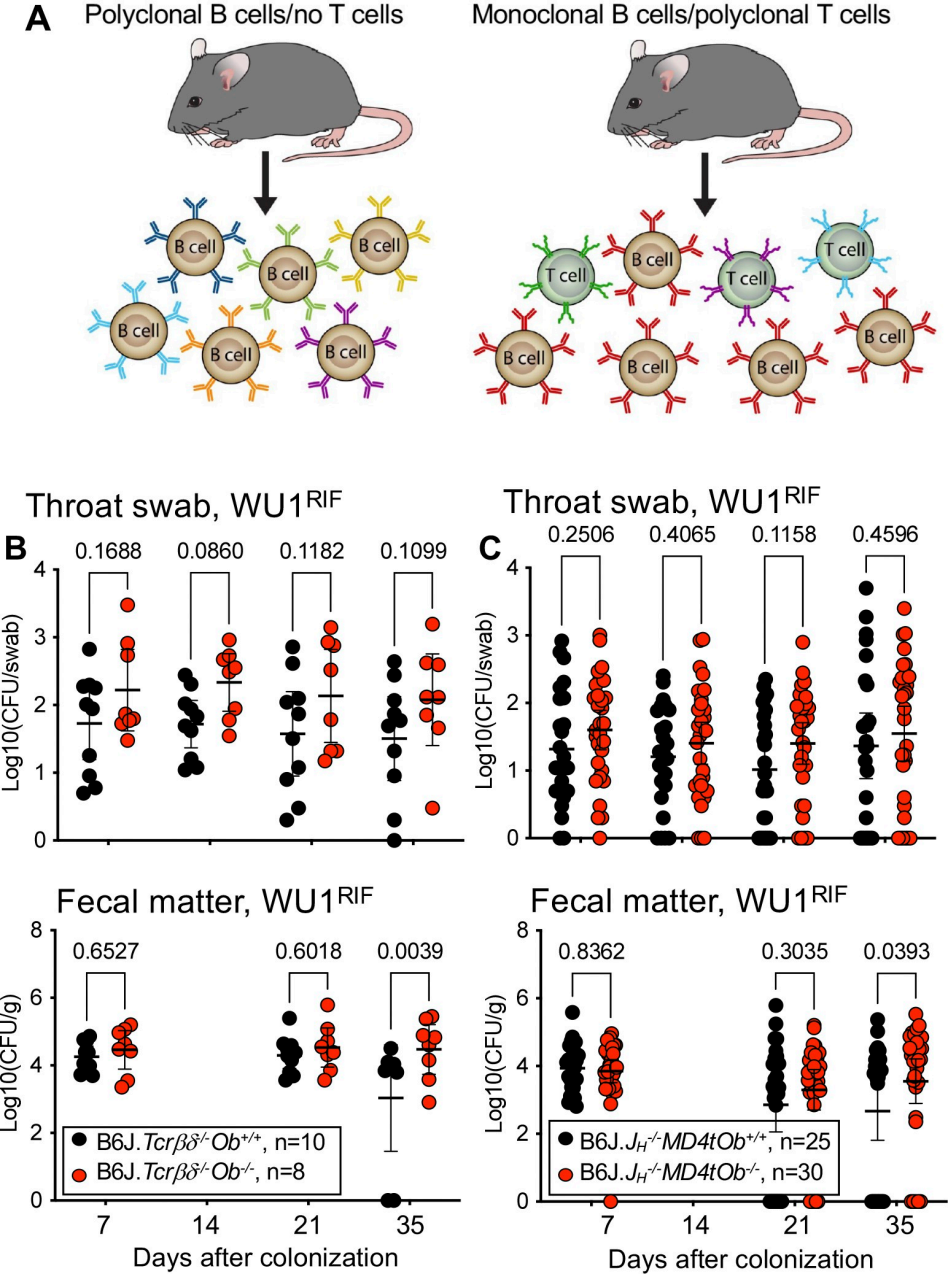
H2-O-deficient mice control *S*. *aureus* by T- and B-cell dependent responses. Eight-week-old *TCRβδ*^*-/-*^*Ob*^+/+^ and *TCRβδ*^*-/-*^*Ob*^*-/-*^ **(A, left)** and *J*_*H*_^*-/-*^*MD4tOb*^+/+^ and *J*_*H*_^*-/-*^*MD4tOb*^*-/-*^ **(A, right)** B6J mice were colonized intranasally with 10^8^ CFUs of WU1^Rif^ and monitored for colonization weekly by plating throat swabs **(B** and **C, top panels)** and fecal matter **(B** and **C, bottom panels)**. Significance was calculated using two-way ANOVA tests with multiple comparisons. Data are presented as median ± 95% confidence interval. n, number of mice used. Mice of different genotypes were co-housed. Males and females were used at 50:50 ratio. CFU, colony forming unit.

Since H2-O functions within the MHC-II antigen presentation pathway, we reasoned that control of staphylococcal species in H2-O-deficient mice is mediated via the adaptive immune response. To address this possibility, *Ob*^*-/-*^ and *Ob*^*+/+*^ mice that either lack T cells or are monoclonal for the B cell receptor (BCR) were generated (Fig 4A). Briefly, to produce T-cell deficient mice, crosses were performed between B6.*Tcrβ*^*-/-*^, B6.*Tcrδ*^*-/-*^, B6.*Ob*^*-/-*^ and wild type (B6.*Ob*^*+/+*^) animals to obtain B6.*Tcrβδ*^*-/-*^ (which lack both αβ and γδ T cells) with and without *Ob*. The absence of T cells does not affect H2-O function as H2-O is expressed only in B, dendritic and thymic epithelial cells [30–33]. To produce BCR monoclonal *Ob*^*-/-*^ and *Ob*^*+/+*^, the B6.*MD4t* transgenic mice, which carry a transgene for the rearranged heavy and light chains of a BCR recognizing hen egg lysozyme [50], were crossed to *Ob*^-/-^ mice and subsequently to mice deficient for the IgH J segment locus (J_H_) [51]. The resulting B6.*J*_*H*_^*-/-*^*MD4tOb*^*-/-*^ and B6.*J*_*H*_^*-/-*^*MD4tOb*^*+/+*^ animals lacked all endogenous B cells and had only B cells of the transgenic origin (Fig 4A; S1A and S1B Fig). T-cell deficient and BCR monoclonal mice with and without *Ob* were colonized with WU1^Rif^ and monitored for the presence of bacteria over time (Fig 4B and 4C). B6.*Ob*^*+/+*^ and B6.*Ob*^*-/-*^ mice were also colonized as control (S2 Fig). As reported in Fig 1, B6.*Ob*^*-/-*^ animals gradually decolonized *S*. *aureus* (S2 Fig) while B6.*Tcrβδ*^*-/-*^*Ob*^*-/-*^ (Fig 4B) and B6.*J*_*H*_^*-/-*^*MD4tOb*^*-/-*^ (Fig 4C) mice maintained colonization as did B6.*Ob*^*+/+*^ (S2 Fig), B6.*Tcrβδ*^*-/-*^ *Ob*^*+/+*^ (Fig 4B) and B6.*J*_*H*_^*-/-*^*MD4t Ob*^*+/+*^ (Fig 4C) mice. These data indicate that *S*. *aureus*-specific T cells and B cells are required for bacterial clearance in H2-O-deficient mice.

### Anti-*S*. *aureus* responses in H2-O-deficient mice are broadly protectives

**Fig 5:**
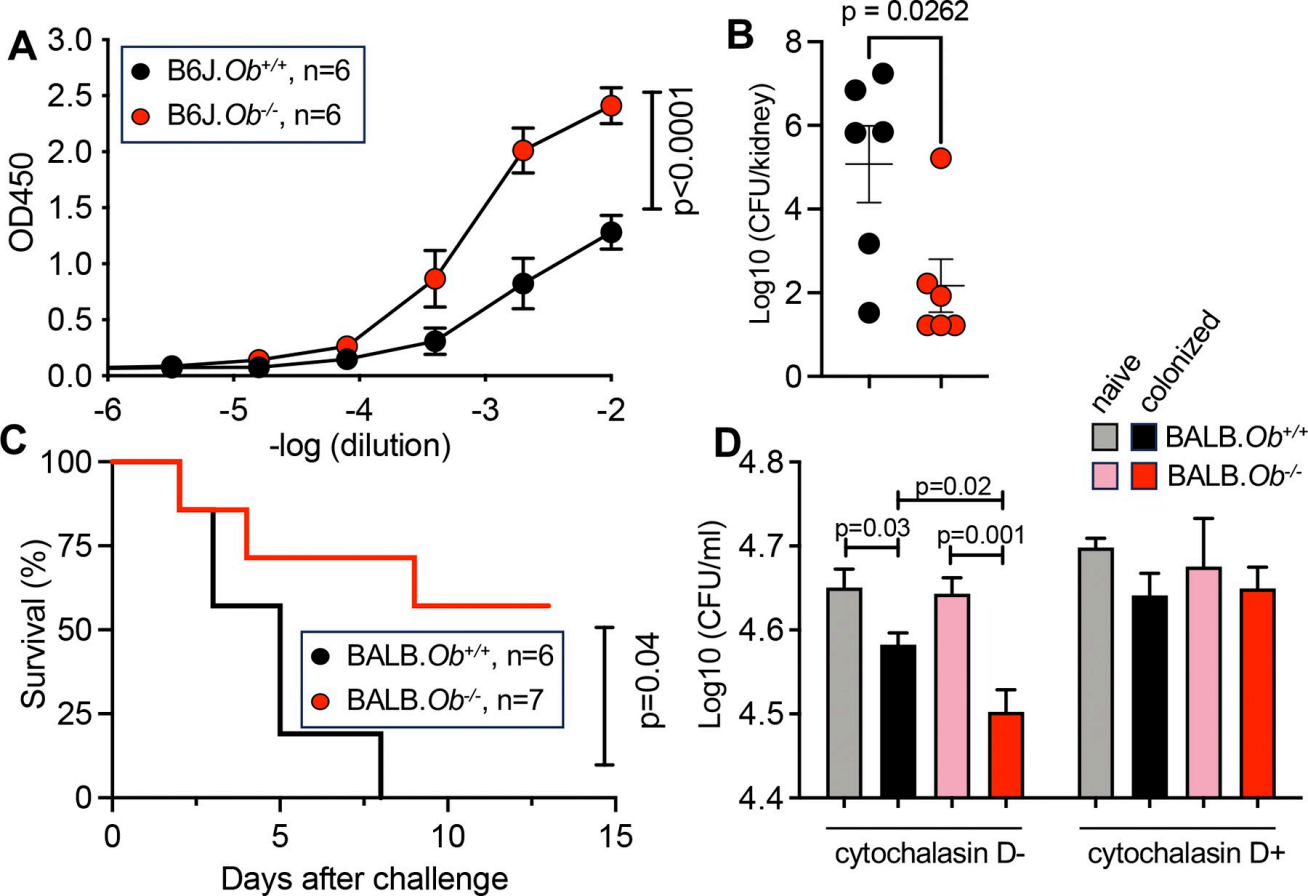
WU1-colonized *Ob*^*-/-*^ mice are protected from a *S*. *aureus* bloodstream challenge. **(A)** Seven weeks after colonization, *Ob*^*+/+*^ and *Ob*^*-/-*^ B6J mice (shown in **Fig 1A**) were bled, and their sera analyzed for *S*. *aureus*-reactive IgGs in ELISA using a Δ*spa*Δ*sbi S*. *aureus* lysate. **(B)** Eight weeks post colonization B6J.*Ob*^*+/+*^ and B6J.*Ob*^*-/-*^ mice shown in **Fig 1** were challenged intravenously with 5×10^6^ CFU of *S*. *aureus* strain USA300 and sacrificed 15 days later to enumerate CFU in kidneys. (**C**) Ten weeks after colonization, BALB/c.*Ob*^*+/+*^ and BALB/c.*Ob*^*-/-*^ mice (shown in **Fig 2**) were challenged intravenously with 1×10^7^ CFU of *S*. *aureus* strain USA300 and monitored over 14 days. Animals that lost more than 20% of their original weight and showed clear signs of disease were removed from the study. (**D**) *S*. *aureus* replication in mouse blood supplemented with pooled sera obtained from naïve (12–15 weeks of age) and colonized BALB/c.*Ob*^*+/+*^ and BALB/c.*Ob*^*-/-*^ mice (9 weeks after colonization, mice shown in **Fig 2**). n = 6–7 mice per groups. Bacterial survival was measured in the presence (+) or absence (-) cytochalasin D. Significance was calculated using two-way ANOVA tests with multiple comparisons **(A)**, unpaired *t* test **(B, D)**, and Mantel-Cox test **(C)**. Data are presented as mean ± SEM **(A**, **B**, **D).** n, number of mice used.

Next, we sought to determine whether WU1 decolonization in *Ob*^*-/-*^ mice correlated with increased pathogen-specific antibody titers. Sera from WU1-colonized B6J.*Ob*^*+/+*^ and B6J.*Ob*^*-/-*^ mice from the experiment shown in Fig 1A, were analyzed for anti-*S*. *aureus* IgGs in ELISA using cellular extracts of a strain lacking *spa* and *sbi* (Δ*spa*Δ*sbi*) to avoid non-specific binding with antibodies [52]. This analysis revealed a significant increase in *S*. *aureus*-specific IgGs in colonized B6J.*Ob*^*-/-*^ compared to B6J.*Ob*^*+/+*^ mice (Fig 5A).

To further assess the protective attribute of *S*. *aureus*-specific IgG responses, *S*. *aureus*-colonized B6J.*Ob*^*-/-*^ and *Ob*^*+/+*^ animals (shown in Fig 1) were subjected to a sublethal intravenous challenge with 5×10^6^ CFU of *S*. *aureus* strain USA300, a methicillin-resistant *S*. *aureus* (MRSA) clone and prevalent cause of community-associated skin and soft tissue and bloodstream infections [53,54]. Similar to humans, bloodstream infection of mice with *S*. *aureus* results in either death or the dissemination to organ tissues and the development of abscess lesions that persist for weeks [1,55,56]. Bloodstream dissemination is measured as bacterial loads in kidneys by plating tissues fifteen days after challenge and recording colony forming units (CFUs) per kidney [55,56]. Bacterial burdens were reduced by approximately 5×10^3^ times in WU1-colonized B6J.*Ob*^*-/-*^ mice as compared to B6J.*Ob*^*+/+*^ (Fig 5B).

BALB.*Ob*^*-/-*^ and BALB.*Ob*^*+/+*^ mice (shown in Fig 2) were also challenged 9 weeks post colonization with what turned out to be a slightly higher dose (1×10^7^ CFU of strain USA300) resulting in the development of acute disease (Fig 5C). None of the *Ob*^*+/+*^ animals survived the intravenous challenge, while ~60% of the *Ob*^*-/-*^ mice did. Further, all surviving animals had recovered a normal health status by day 11 post infection (Fig 5C).

To rule out the possibility that the loss of *Ob* alone might confer resistance to bloodstream dissemination, groups of naïve B6J.*Ob*^*+/+*^ and B6J.*Ob*^*-/-*^ and mice (S3A Fig) and BALB.*Ob*^*+/+*^ and BALB.*Ob*^*-/-*^ animals (S3B Fig) were also challenged with 5×10^6^ CFU of strain USA300 and killed 15 days later. *Ob*^*-/-*^ and *Ob*^*+/+*^ mice of both genetic backgrounds displayed similar bacterial burdens in kidney tissues (S3 Fig).

The data presented so far suggest that the *S*. *aureus*-specific antibodies generated in colonized *Ob*^*-/-*^ mice have improved functional activity compared to *S*. *aureus*-specific antibodies produced by colonized *Ob*^*+/+*^ mice. Complement- and Fc receptor-mediated phagocytosis are two major mechanisms known to control *S*. *aureus* in an antibody-dependent manner [57]. The presence of opsonophagocytic antibodies in sera can be tested using a whole blood killing assay [57]. Thus, sera from colonized BALB.*Ob*^*-/-*^ and BALB.*Ob*^*+/+*^ (shown in Fig 2) as well as sera from naïve BALB.*Ob*^*-/-*^ and BALB.*Ob*^*+/+*^ (control) were added to anti-coagulated, freshly drawn blood of naïve C57BL/6.μMT animals that lack mature B cells and thus, most immunoglobulins [58,59] and then incubated with *S*. *aureus* for 30 min. Next, samples were treated with saponin and streptokinase to lyze host cells as well as liberate extracellular bacteria that agglutinate in fibrin to escape opsnophagpcytic killing [60] and plated to enumerate *S*. *aureus* CFUs. A statisitcal reduction in bacterial CFUs was observed in samples incubated with sera from both colonized BALB.*Ob*^*+/+*^ and BALB.*Ob*^*-/-*^ as compared to the isogenic naïve animals (Fig 5D). However, sera of colonized BALB.*Ob*^*-/-*^ had superior killing activity as compared to all other sera. Bacterial killing was not observed when blood had been pretreated with cytochalasin D, a cell-permeable inhibitor of actin polymerization (Fig 5D). Thus, exposure to *S*. *aureus* elicits opsonophagocytic antibodies more effectively in *Ob*-deficient compared to *Ob*-sufficient animals.

## Discussion

All humans are exposed to *S*. *aureus* at birth and most remain colonized through life while some may be intermittently colonized [5,6,61]. Healthy carriage represents a major cause for infection through self-inoculation [7–10]. *S*. *aureus* is a highly pathogenic organism and its wide distribution in human populations suggest that not all individuals may be similarly susceptible to infection. Because strains of *S*. *aureus* are adaptable to other species, inquiries on susceptibility can be experimentally addressed in animals. For example, mice and sheep from different genetic backgrounds were found to display variable susceptibilities to *S*. *aureus* infection [62–64]. In humans, correlations may be primarily drawn between genetic conditions and increased susceptibility. It has long been recognized that primary immunodeficiencies such as chronic granulomatous disease (CGD) of childhood predispose the host to severe and recurrent bacterial infections [65–67]. Polymorphonuclear neutrophils play a key role in defending against *S*. *aureus* [68,69] and the defective NADH oxidase accounts for the impaired bactericidal activity of CGD neutrophils [65,66]. Other single-gene inborn errors of immunity have also been found to predispose individuals to *S*. *aureus* infections and include congenital neutropenia, leukocyte adhesion deficiency, and germline mutations of the TLR and IL-1R pathways [70]. Inborn errors of immunity have also been found to exacerbate disease. For example, OTULIN haploinsufficiency has been shown to enhance the cytotoxicity of the staphylococcal virulence factor α-toxin [71]. However, as these mutations are very rare, they do not account for the large number of staphylococcal infections that affect otherwise healthy individuals [72]. Single nucleotide polymorphism analyses and genome wide association studies have also been applied to identify genetic markers that may influence *S*. *aureus* colonization focusing on host adhesive properties, recognition (mannose-binding lectin, TLRs, NLRs and PRRs) or eradication (antimicrobial peptides, cytokines) of the pathogen [73]. Genetic variations of mannose-binding lectin, TLR2 and TLR4 were found to correlate with increased nasopharyngeal colonization in infants [74]. It has also been reported that healthy *S*. *aureus* carriers produce more LL-37, a cathelicidin antimicrobial peptide, than non-carriers but a genetic basis was not revealed [75]. Thus, known and unknown genetic variations could render a host more resistant to *S*. *aureus* colonization.

The contribution of H2-O, an inhibitor of H2-M that limits the number of high affinity peptides presented by MHC-II [43], was examined in a mouse model of *S*. *aureus* colonization. We find that mouse lacking H2-O become decolonized in a B- and T-cell-dependent manner. Further, animals lacking H2-O that had been colonized with *S*. *aureus* were significantly better protected against subsequent bloodstream challenge with *S*. *aureus* as compared to colonized wild type animals or naïve H2-O-sufficient and -deficient animals. This protection correlated with increased anti-*S*. *aureus* serum responses and enhanced opsonophagocytic activity of *S*. *aureus*-specific antibodies as revealed in a whole blood killing assay. Thus, loss of H2-O represents a gain of function with respect to control of the pathogen. Increased resistance to *S*. *aureus* in absence of H2-O was observed in both B6J and BALB/cJ mice with MHC haplotypes H2^b^ and H2^d^, respectively, suggesting that protection may be achieved upon recognition of distinct antigens. Antibody-mediated decolonization promoted in H2-O animals mirrors decolonization observed with *S*. *aureus* lacking Staphylococcal protein A (WU1Δ*spa*) [3]. SpA is a B cell superantigen that induces the production of non-specific IgGs that dilute the activity of protective antibodies [19,20,76]. Thus, lack of H2-O in the host or lack of SpA in the pathogen result in protective antibody responses that are key to clearing this mucosal pathogen. Deconvoluting the nature of such responses may prove informative for the identification of optimal epitopes for vaccine designs.

We also observe that H2-O-deficient animals clear *S*. *xylosus*. *S*. *xylosus* generally inhabits the skin and mucous membranes of birds and mammals including laboratory mice [77,78]. Like *S*. *aureus*, *S*. *xylosus* has been shown to cause invasive disease in otherwise healthy carrier mice albeit that the mechanisms promoting colonization or infection have not been investigated [79]. We surmise that loss of *S*. *xylosus* colonization in H2-O-deficient animals is also the result of increased antibody responses.

## Materials and methods

### Ethics statement

Animal research was performed in accordance with institutional guidelines following experimental protocol review, approval, and supervision by the Institutional Animal Care and Use Committee at The University of Chicago. Experiments with *S*. *aureus* were performed in Biosafety Level 2 containment upon review by The University of Chicago Institutional Biosafety Committee.

### Mice

C57BL/6J (stock #000664), BALB/cJ (stock #000651) B6.129P2-Tcrb^tm1Mom^/J (stock #002118), B6.129P2-Tcrd^tm1Mom^/J (stock #002120), and C57BL/6-Tg(IghelMD4)4Ccg/J (stock #002595), B6J.μMT (stock 002288) mice were purchased from The Jackson Laboratory. B6J.*Ob*^*-/-*^ [29] and BALB.*Ob*^*-/-*^ [80] mice on the C57BL6/J and BALB/cJ background, were from our laboratory collections. B6J.J_H_^-/-^ mice [51] were a gift from Dr. Albert Bendelac (The University of Chicago). All animals were bred at the University of Chicago in a pathogen-free animal facility. For blood draw, intranasal inoculation, and intravenous infection, animals were anesthetized with a cocktail of ketamine-xylazine (50 to 65 and 3 to 6 mg/kg).

### FACS

Splenocytes were Fc-blocked and stained with the following monoclonal antibodies (mAb) α-CD19-PE (clone 6D5, BioLegend), α-IgM^b^-FITC (clone AF6-78, BioLegend, for detection of MD4 transgenic B cells of the BALB/cJ origin) and α-IgM^a^-APC (clone MA-69, for detection of B cells of the B6J origin). Dead cells were excluded using propidium iodide.

### Bacterial strains and growth conditions

*S*. *aureus* strains WU1, WU1^Rif^, USA300 (LAC) and the Δ*spa*Δ*sbi* variant were from our laboratory collection and propagated in tryptic soy broth or tryptic soy agar at 37°C. Animal swabs and fecal samples were plated on mannitol salt agar at 37°C. For animals colonized with WU1^Rif^ or infected with USA300, test samplings were plated on tryptic soy agar containing 100 μg/ml rifampicin or 50 μg/ml kanamycin, respectively.

### Preparation of bacterial cultures and extracts

Bacterial inocula for animal colonization and infection were prepared as follows. For each experiment, an overnight culture grown from a single colony in tryptic soy broth was diluted 100 times and grown to absorbance at 600 nm (*A*_600_) of approximately ~1. Cultures were spun to sediment bacteria and cells were washed by resuspension in an equal volume of PBS. After a second sedimentation, cells were resuspended in PBS at ~10^10^ CFU/ml and 10 μl (10^8^ CFU) were used for intranasal inoculation. For intravenous challenge, bacteria were resuspended at a concentration of ~5–10×10^7^ CFU/ml and 100 μl of this suspension was injected in animals. For whole blood killing assays, bacteria were resuspended at a concentration of ~1×10^6^ CFU/ml. For ELISA experiments, cultures grown to *A*_600_ of ~0.5 were subjected to 20 μg/mL lysostaphin treatment for 30 min at 37°C, followed by 10% trichloroacetic acid precipitation on ice for 1 h. Samples were centrifuged for 10 min at 20,000 × g, and precipitates washed with ice-cold acetone, allowed to dry, and suspended in PBS.

### Animal experiments

Following intranasal inoculation with bacteria, mice were monitored daily and throat swabs and fecal pellets were obtained in weekly intervals. Throat swabs were performed with CONSTIX swabs (pointed 0.08x0.30 in, cat# SC-4). Throats of mice were swabbed to a depth of approximately 17mm and streaked across agar plates. Fecal pellets were collected into sterile pre-weighed tubes, weighed, and homogenized in 500 μl of PBS. Following intravenous infection with bacteria, animals were monitored for signs of illness for up to 15 days. To measure bacterial loads in tissues, mice were sacrificed and kidneys were homogenized in PBS with 0.1% Triton X-100 and serially diluted prior to plating.

### Whole blood killing assay

Tubes containing 300 μl of freshly drawn B6J.μMT mouse blood anticoagulated with heparin (10 unit/ml) were pre-incubated for 10 min with 20μM cytochalasin D in DMSO or vehicle control (DMSO). Each tube received 15 μl of sera pooled from either naïve or colonized BALB.*Ob*^*-/-*^ and BALB.*Ob*^*+/+*^ mice. Reactions were started upon addition of 15 μl of PBS containing 1.5×10^4^ CFU of *S*. *aureus* and proceeded for 30 min at 37°C with rotation. 300 μl SK buffer containing 2% saponin, 200U/ml streptokinase, 1mg/ml trypsin, 20 μg/ml DNase, 100 μg/ml RNase A was added to each sample for 10 min at 37°C prior to plating for CFU enumeration. This step was performed to liberate bacteria from fibrin agglutinates [60]. The experiment was repeated an additional time and all assays were performed in duplicate.

### Enzyme-linked immunosorbent assay (ELISA)

Microtiter plates (NUNC MaxiSorp) were coated with 1 μg/ml of Δ*spa*/Δ*sbi* bacterial extracts in 0.1 M carbonate buffer (pH 9.5) at 4°C overnight. Wells were blocked, incubated with serial dilutions of mouse sera prior to incubation with 1 mg/ml horseradish peroxidase (HRP)-conjugated goat anti-mouse antibodies (Fisher Scientific) and developed using OptEIA reagent (BD Biosciences). Experiments were performed in triplicate to calculate averages and standard error of the mean and repeated for reproducibility.

### Statistical analyses

All experiments were performed at least twice. For experiments with repeated measures, data was plotted as median ± 95% confidence interval and analyzed using two-way analysis of variance (ANOVA) with multiple-comparison tests (GraphPad Software). For experiments show in Figs 3A, 3C, 5B and 5D with only two groups and a single time point, data was analyzed with unpaired *t* test. The Mantel-Cox test was used to analyze data in Fig 5A and 5C.

## Supporting information

S1 Fig Flow cytometric gating strategies to stain B cells in wild type and MD4 transgenic JH-/- mice. Red blood cells were lysed to prepare single cell suspensions of splenocytes that were stained with monoclonal antibodies specific for CD19 to mark B cells (α-CD19-PE) and for IgM allotype ‘a’ (α-IgM^a^-APC) and ‘b’ (α-IgM^b^-FITC). Staining with α-IgM^b^-FITC identifies B cells carrying endogenous B6J heavy chains, while staining with α-IgM^a^-APC identifies cells bearing the MD4 transgene, which is of the BALB/cJ origin. BALB/cJ and B6J mice were used as controls. **(A)** Representative scatter plots demonstrating gating strategy. **(B)** Percent of B cells of B6J origin in 3 non-transgenic B6J and three J_H_^-/-^MD4t B6J mice. t, transgenic. n/t, non-transgenic.
(TIF)

S2 Fig H2-O-deficient mice control *S*. *aureus*. Eight-week-old *Ob*^+/+^ and *Ob*^*-/-*^ B6J mice (control for mice shown in Fig 4) were colonized intranasally with 10^8^ CFUs of WU1^Rif^ and monitored for colonization weekly by plating throat swabs **(A)** and fecal matter **(B)**. Significance was calculated using two-way ANOVA tests with multiple comparisons. Data are presented as median ± 95% confidence interval. n, number of mice used. Mice of different genotypes were co-housed. Males and females were used at 50:50 ratio. CFU, colony forming unit.
(TIF)

S3 Fig Naïve *Ob*^*-/-*^ mice are not protected from a *S*. *aureus* bloodstream challenge. Naïve B6J.*Ob*^*+/+*^ and B6J.*Ob*^*-/-*^ **(A)** and naïve BALB/c.*Ob*^*+/+*^ and BALB/c.*Ob*^*-/-*^ mice **(B)** were challenged intravenously with 5×10^6^ CFU of *S*. *aureus* strain USA300 at 8 weeks of age and sacrificed 15 days later to enumerate CFU in kidneys. Significance was calculated using unpaired *t* test. Data are presented as mean ± SEM. n, number of mice used. Males and females were used at 50:50 ratio.
(TIF)
