# Supplementary figures and images for: Deficiency in non-classical major histocompatibility class II-like molecule, H2-O confers protection against Staphylococcus aureus in mice
Source: PLoS Pathog. 2024 Jun 6;20(6):e1012306. doi: 10.1371/journal.ppat.1012306 (PMC11185455; doi:10.1371/journal.ppat.1012306)

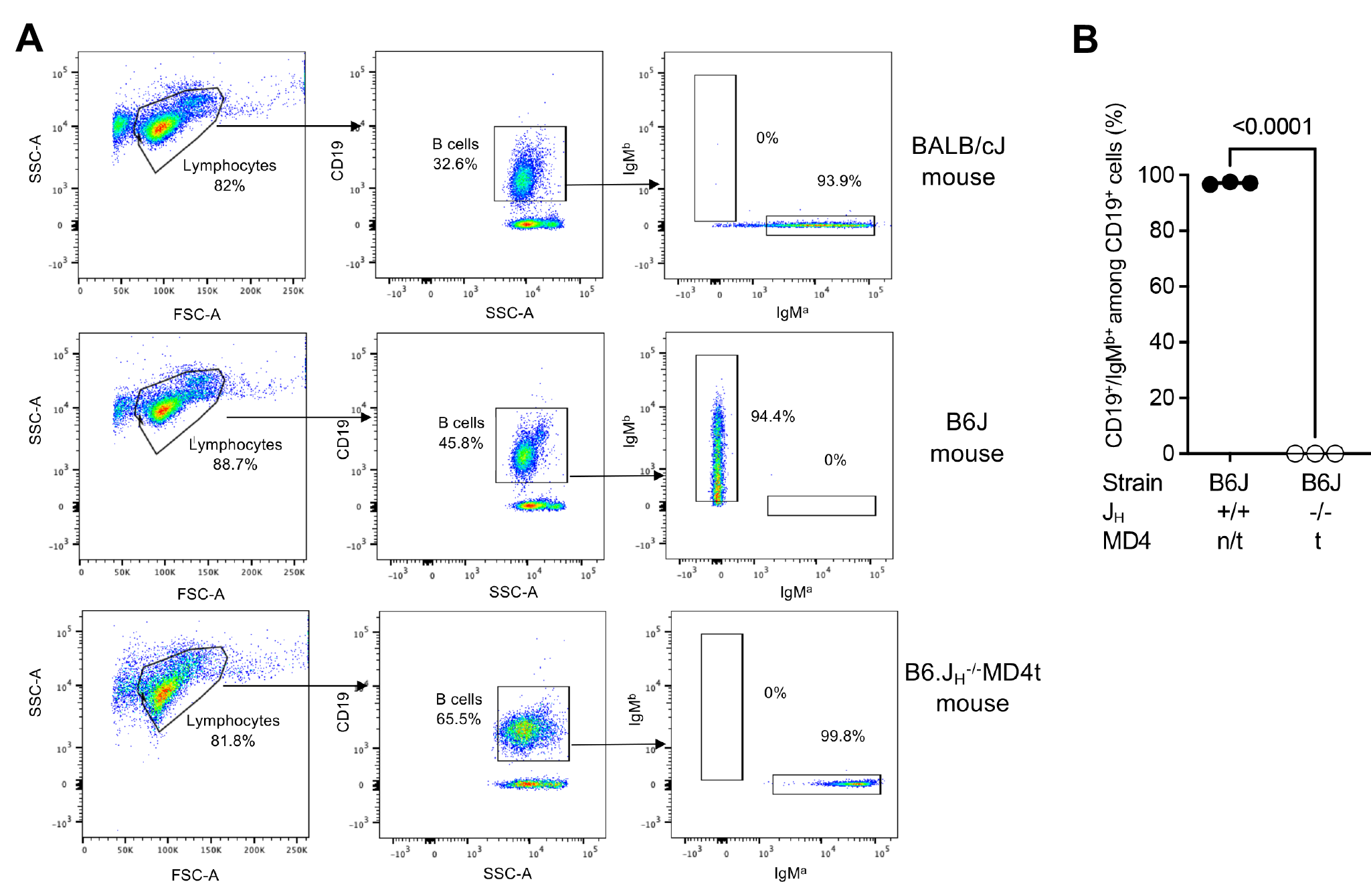

Supplement: S1 Fig — Red blood cells were lysed to prepare single cell suspensions of splenocytes that were stained with monoclonal antibodies specific for CD19 to mark B cells (α-CD19-PE) and for IgM allotype ‘a’ (α-IgMa-APC) and ‘b’ (α-IgMb-FITC). Staining with α-IgMb-FITC identifies B cells carrying endogenous B6J heavy chains, while staining with α-IgMa-APC identifies cells bearing the MD4 transgene, which is of the BALB/cJ origin. BALB/cJ and B6J mice were used as controls. (A) Representative scatter plots demonstrating gating strategy. (B) Percent of B cells of B6J origin in 3 non-transgenic B6J and three JH-/-MD4t B6J mice. t, transgenic. n/t, non-transgenic. (TIF) [file ppat.1012306.s001.tif]

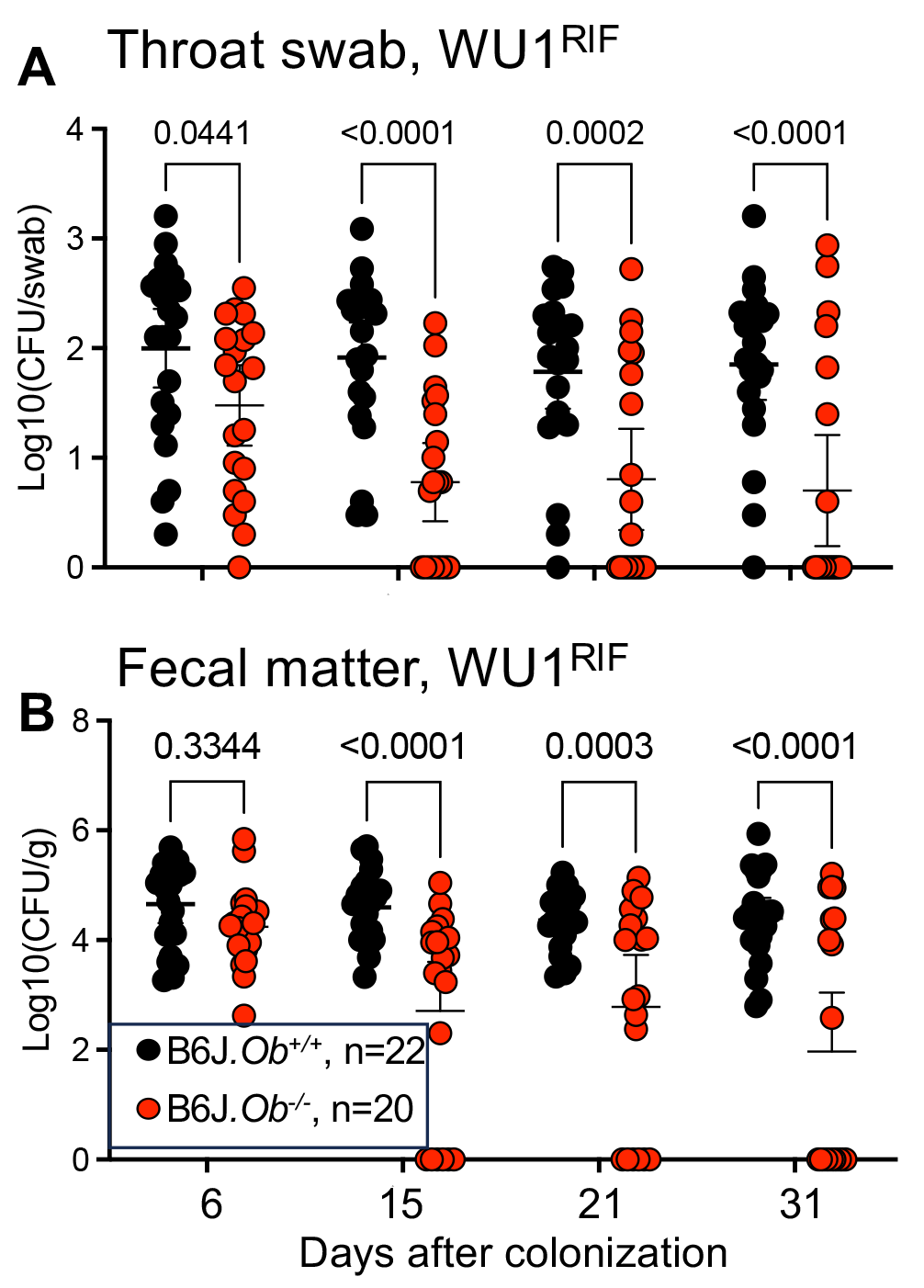

Supplement: S2 Fig — Eight-week-old Ob+/+ and Ob-/- B6J mice (control for mice shown in Fig 4) were colonized intranasally with 108 CFUs of WU1Rif and monitored for colonization weekly by plating throat swabs (A) and fecal matter (B). Significance was calculated using two-way ANOVA tests with multiple comparisons. Data are presented as median ± 95% confidence interval. n, number of mice used. Mice of different genotypes were co-housed. Males and females were used at 50:50 ratio. CFU, colony forming unit. (TIF) [file ppat.1012306.s002.tif]

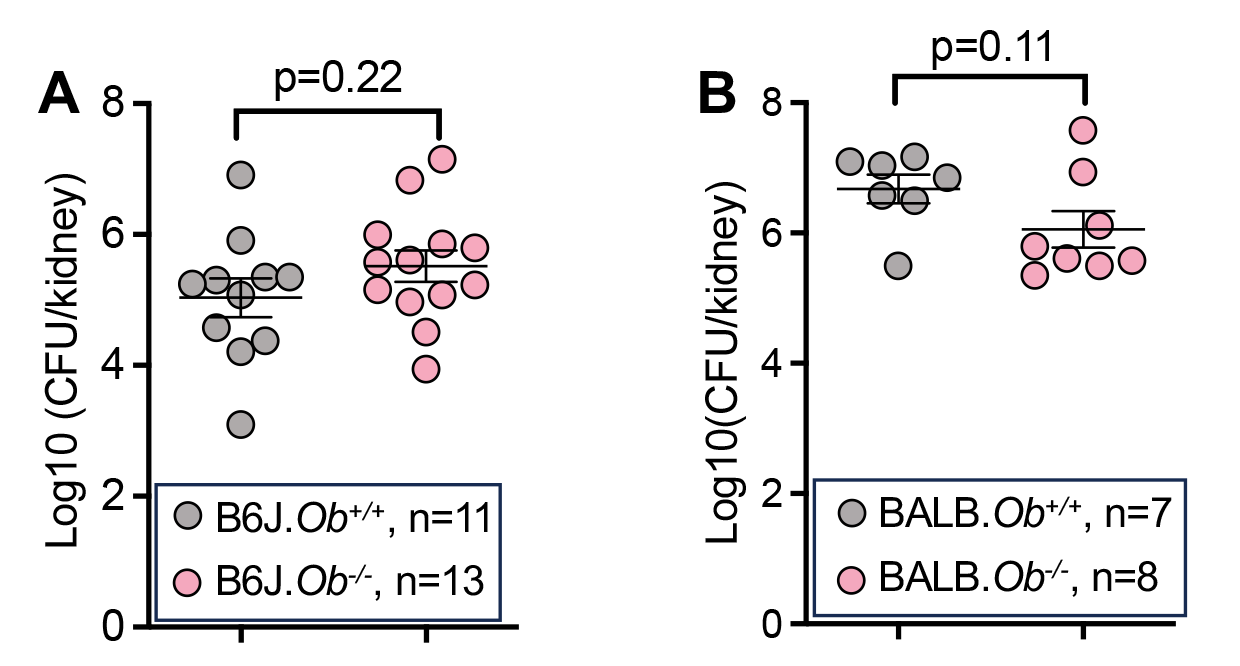

Supplement: S3 Fig — Naïve B6J.Ob+/+ and B6J.Ob-/- (A) and naïve BALB/c.Ob+/+ and BALB/c.Ob-/- mice (B) were challenged intravenously with 5×10^6^ CFU of S. aureus strain USA300 at 8 weeks of age and sacrificed 15 days later to enumerate CFU in kidneys. Significance was calculated using unpaired t test. Data are presented as mean ± SEM. n, number of mice used. Males and females were used at 50:50 ratio. (TIF) [file ppat.1012306.s003.tif]
